# Case Report: m.13513 G>A Mutation in a Chinese Patient With Both Leigh Syndrome and Wolff-Parkinson-White Syndrome

**DOI:** 10.3389/fped.2021.700898

**Published:** 2021-07-01

**Authors:** Jian-Min Liang, Cui-Juan Xin, Guang-Liang Wang, Xue-Mei Wu

**Affiliations:** ^1^Department of Pediatric Neurology of Jilin University, Changchun, China; ^2^Jilin Provincial Key Laboratory of Pediatric Neurology, Changchun, China; ^3^Department of Cardiology, Dalinghe Hospital of Far Eastern Horizon, Linghai, China

**Keywords:** Leigh syndrome, 13513 mutation, Wolff-Parkinson-White syndrome 2, neurology, pediatric

## Abstract

A number of causative mutations in mitochondrial and nuclear DNA have been identified for Leigh syndrome, a neurodegenerative encephalopathy, including m. 8993 T>G, m.8993 T>C, and m.3243A>G mutations in the *MTATP6, MTATP6*, and *MT-TL1* genes, respectively, which have been reported in Leigh syndrome patients in China. The m.13513 G>A mutation has been described only a few times in the literature and not previously reported in China. Here we report the case of a 15-month-old boy who presented with ptosis and developmental delay and was diagnosed with Leigh syndrome and well as Wolff-Parkinson-White (WPW) syndrome. The m.13513 G>A mutation was found in DNA from blood. He was intubated due to respiratory failure and died at 23 months of age. The m.13513 G>A mutation in the *ND5* gene of mitochondrial DNA is associated with Leigh syndrome and WPW syndrome; however, this is the first report of this mutation in a patient in China, highlighting the geographical and racial variability of Leigh syndrome.

## Introduction

Leigh syndrome is a neurodegenerative disorder characterized by bilateral symmetrical necrotic lesions in the basal ganglia and brainstem that is typically identified within the first year of life (1). In China, a common causative mutation for Leigh syndrome is the *SURF1* mutation, and m.8993 T>G, m.8993 T>C, and m.3243A>G mutations in the *MTATP6, MTATP6*, and *MT-TL1* genes also have been identified as causative mutations in Chinese patients with Leigh syndrome (2). Another mutation, the m.13513 G>A mutation, is more commonly associated with MELAS (Mitochondrial Encephalopathy, Lactic Acidosis, and Stroke-like episodes) (3, 4), but ~7% of Leigh syndrome cases in Western countries are estimated to be caused by the m.13513 G>A mutation (5). However, this mutation has not previously been reported in China in association with Leigh syndrome. Here we present the case of a 15-month-old boy in whom the G-to-A transition at nucleotide 13513 of the mitochondrial *ND5* gene was identified and who died at the age of 23 months. As the first report of this mutation in China, it highlights the geographical and racial variability of Leigh syndrome.

## Case Description

**Figure 1:**
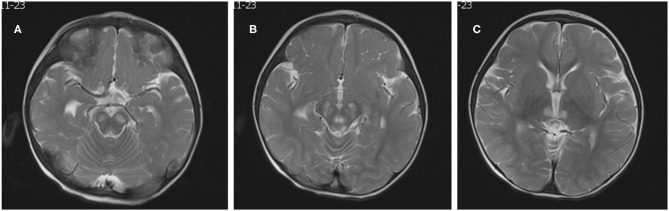
Neuroradiological features. Brain MRI showed bilateral, symmetric signal abnormalities in the basal ganglia, and brain stem (**A–C**: T2-weighted images).

**Figure 2:**
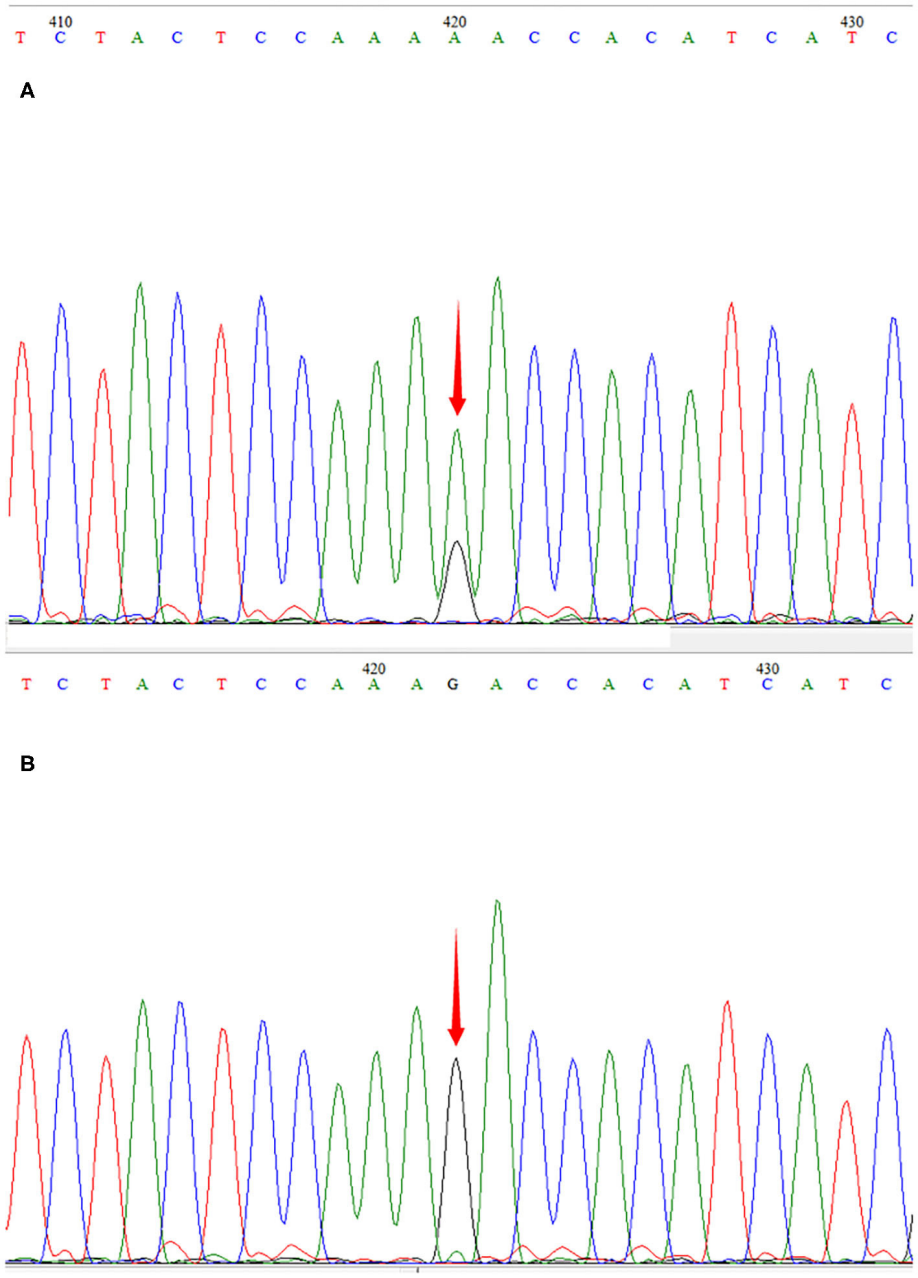
Sequencegrams. m.13513G>A was identified in the DNA of the patient **(A)**. The mutation was not found in DNA samples derived from his mother **(B)**.

The patient was a 15-month-old boy. He was delivered by cesarean section because of a low level of amniotic fluid at 39 weeks of gestational age, with a birth weight of 2,540 g. To the time of presentation, he had experienced poor body weight gain and delayed gross motor development. He could not sit up without support. His mother was clinically normal. At the age of 15 months, he was brought to our outpatient clinic for ptosis and developmental regression. On admission, his body weight was 8.1 kg (normal weight range 9.8–12.2 kg) and body length was 75.5 cm (normal body length range 78.2–84 cm). He could say simple words such as “papa” and “mama.” Bilateral ptosis was found, but no obvious optic atrophy was detected by an ophthalmologist. Bilateral knee jerk was also exaggerated. Initial laboratory studies revealed normal complete blood counts as well as liver aspartase, blood urea nitrogen, creatinine, and electrolyte levels within normal ranges. His serum lactate level was elevated to 3.0 mmol/L (normal <2.2 mmol/L) and the cerebrospinal fluid lactate level was elevated to 2.9 mmol/L (normal <2.7 mmol/L). WPW syndrome was revealed by electrocardiography with delta waves indicating left bundle branch block. The results of electrocardiography and a Holter electrocardiography were normal for both parents. Cardiac magnetic resonance imaging (MRI) was normal. Brain MRI showed bilateral and symmetrical signal abnormalities in the thalami and midbrain (Figure 1). The clinical phenotype combined with the elevated lactate level and cerebral lesions strongly suggested a mitochondrial encephalopathy or Leigh syndrome. DNA sequence analysis revealed that the patient had a G-to-A transition at mitochondrial DNA nt 13513 in blood specimens, resulting in an amino acid change of Asp to Asn in the mitochondrial *ND5* gene by LR-PCR and next-generation sequencing (NGS, Figure 2). The mutation ratio was 86%. The patient's mother either did not carry the mutations in blood specimens. There was no positive result from array comparative genomic hybridization (CGH) including the *PRKAG2* gene in the family trio. Coenzyme Q10 (90 mg/d), vitamin B complex (100 mg/d), and carnitine (300 mg/d) were prescribed following the diagnosis of Leigh syndrome. The patient experienced mild improvements in ptosis. However, at the age of 23 months, he was intubated for respiratory failure caused by pneumonia and died.

## Discussion

The incidence of Leigh syndrome [Mendelian Inheritance in Man (MIM) 25600] is 0.025‰ (6), and to date, over 75 pathogenic genetic mutations have reported for Leigh syndrome (7). However, the incidences of the mutations appear to differ among specific populations.

The reported patient was diagnosed with Leigh syndrome after the onset of ptosis and developmental regression and the m.13513 G>A mutation was identified. The clinical phenotype of the G13513A mutation has significant heterogeneity. Adult onset is not uncommon, and it may appear as MELAS-Leigh syndrome or alternatively. It may be accompanied by optic atrophy, cataract, deafness and cardiac conduction dysfunction. Unlike most cases of Leigh syndrome in infants with ataxia, convulsion, peripheral nerve, and spinal cord injury, and loss of tendon reflex, the clinical phenotype in this case at presentation included only ptosis, developmental delay and Wolff-Parkinson-White (WPW) syndrome, which has never been described in China. Brain MRI changes showed typical symmetrical lesions in the basal ganglia and brain stem consistent with Leigh syndrome. Additionally, WPW syndrome was revealed on the patient's electrocardiogram. While the m.13513 G>A mutation first found in association with MELAS (3, 4), a recent report also described the m.13513 G>A mutation in cases of Leigh syndrome, especially in association with WPW syndrome (8). Leigh syndrome is commonly caused by both nuclear and mitochondrial DNA mutations of respiratory chain complex I genes (9). Kirby et al. reported that the m.13513 G>A mutation causes a complex I defect even at a low mutation level (10). Consistent with the present case, Wang et al. reported that the m.13513 G>A mutation plays an important role in Leigh syndrome and WPW syndrome, with cardiovascular involvement being common (8). Thus, screening for m.13513 G>A mutation should be applied in patients with Leigh syndrome and WPW syndrome.

While the m.13513 G>A mutation in Leigh syndrome in association with WPW syndrome has been described a few times in the literature, this mutation has not been reported previously in a patient in China. Yang et al. reported 65 unrelated patients who were hospitalized and diagnosed with Leigh syndrome over a 12-year period. In their study, the clinical diagnosis of Leigh syndrome was based on clinical findings and the typical neuroradiologic findings. In 30.8% of the reported cases, the diagnosis was confirmed by genetic analysis and autopsy. *SURF1* mutations were identified in 8 (12.3%) families by DNA sequencing. The m.8344 A>G mutation was found in two cases, and the m.8993 T> G, m.8993 T>C, and m.3243A>G mutations were found in three cases each. Thus, in China, the *SURF1* mutation has been the most commonly found mutation in Leigh syndrome (2). The present case is the first reported identification of the m.13513 G>A mutation in a patient with Leigh syndrome in China, and it is notable that it was found in association with WPW syndrome.

In conclusion, while Leigh syndrome has a highly variable genetic phenotype, the present case confirms the association of the m.13513 G>A mutation with both Leigh syndrome and WPW syndrome, which is the first report of this mutation in China. Therefore, the m.13513 G>A mutation should be considered in Chinese patients diagnosed with Leigh syndrome, especially those also diagnosed with WPW syndrome, highlighting the geographical, and racial variability of Leigh syndrome.

## Data Availability Statement

The raw data supporting the conclusions of this article will be made available by the authors, without undue reservation.

## Ethics Statement

This study was approved by the Ethics Committee of 1st hospital of Jilin University. Written informed consent to participate in this study was provided by the participants' legal guardian/next of kin.

## Author Contributions

J-ML was a major contributor in writing the manuscript. C-JX collected the patient data. G-LW analyzed the patient data. X-MW revised the manuscript. All authors read and approved the final manuscript.

## Conflict of Interest

The authors declare that the research was conducted in the absence of any commercial or financial relationships that could be construed as a potential conflict of interest.

## References

[1] Lake NJ Bird MJ Isohanni P Paetau A. Leigh syndrome: neuropathology and pathogenesis. J Neuropathol Exp Neurol. (2015) 74:482–92. 10.1097/nen.0000000000000195 25978847

[2] Yang YL Sun F Zhang Y Qian N Yuan Y Wang ZX. Clinical and laboratory survey of 65 Chinese patients with Leigh syndrome. Chin Med J. (2006) 119:373–7. 16542579

[3] Hsieh YT Yang MT Peng YJ Hsu WC. Central retinal vein occlusion as the initial manifestation of LHON / MELAS overlap syndrome with mitochondrial DNA G13513A mutation–case report and literature review. Ophthalmic Genet. (2011) 32:31–8. 10.3109/13816810.2010.531880 21174521

[4] Folmes CD Martinez-Fernandez A Perales-Clemente E Li X McDonald A Oglesbee D. Disease-causing mitochondrial heteroplasmy segregated within induced pluripotent stem cell clones derived from a patient with MELAS. Stem Cells. (2013) 31:1298–308. 10.1002/stem.1389 23553816 PMC3706526

[5] Chol M Lebon S Bénit P Chretien D de Lonlay P Goldenberg A. The mitochondrial DNA G13513A MELAS mutation in the NADH dehydrogenase 5 gene is a frequent cause of Leigh-like syndrome with isolated complex I deficiency. J Med Genet. (2003) 40:188–91. 10.1136/jmg.40.3.188 12624137 PMC1735406

[6] Rahman S Blok RB Dahl HH Danks DM Kirby DM Chow CW. Leigh syndrome: clinical features and biochemical and DNA abnormalities. Ann Neurol. (1996) 39:343–51. 10.1002/ana.410390311 8602753

[7] Lake NJ Compton AG Rahman S Thorburn DR. Leigh syndrome: one disorder, more than 75 monogenic causes. Ann Neurol. (2016) 79:190–203. 10.1002/ana.24551 26506407

[8] Wang SB Weng WC Lee NC Hwu WL Fan PC Lee WT. Mutation of mitochondrial DNA G13513A presenting with Leigh syndrome, Wolff-Parkinson-White syndrome and cardiomyopathy. Pediatr Neonatol. (2008) 49:145–9. 10.1016/s1875-9572(08)60030-3 19054921

[9] Shigemi R Fukuda M Suzuki Y Morimoto T Ishii E. L-arginine is effective in stroke-like episodes of MELAS associated with the G13513A mutation. Brain Dev. (2011) 33:518–20. 10.1016/j.braindev.2010.07.013 20832210

[10] Kirby DM Boneh A Chow CW Ohtake A Ryan MT Thyagarajan D. Low mutant load of mitochondrial DNA G13513A mutation can cause Leigh's disease. Ann Neurol. (2003) 54:473–8. 10.1002/ana.10687 14520659

